# Supplementary material for: Genetic and Molecular Characterization of H9c2 Rat Myoblast Cell Line
Source: Cells. 2025 Mar 28;14(7):502. doi: 10.3390/cells14070502 (PMC11988023; doi:10.3390/cells14070502)
Supplement: Supplementary file 1 [file cells-14-00502-s001.zip › Supplemental Material.pdf]

Supplements

Genetic Characterization of Embryonic Heart Rat H9c2 Cells: Insights into Cardiac Myoblast Function and Stability

Thomas Liehr [1,*], Stefanie Kankel [1], Eva Miriam Buhl [2], Heidi Noels[3,4], Sarah K. Schröder-Lange [5], and Ralf Weiskirchen ,[5*]

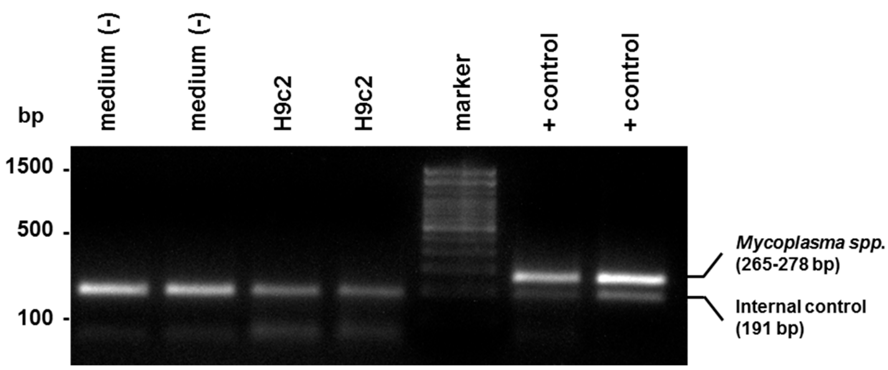

Figure S1. Testing for *Mycoplasma spp.* infection in H9c2 cells using the Venor®GeM OneStep PCR detection kit. The positive kit control displayed the expected 265-278 bp fragment, but this band was not detected in the supernatant from cultured H9c2 cells. However, the internal PCR control band (191 bp) was present in all lanes, confirming a true negative result.

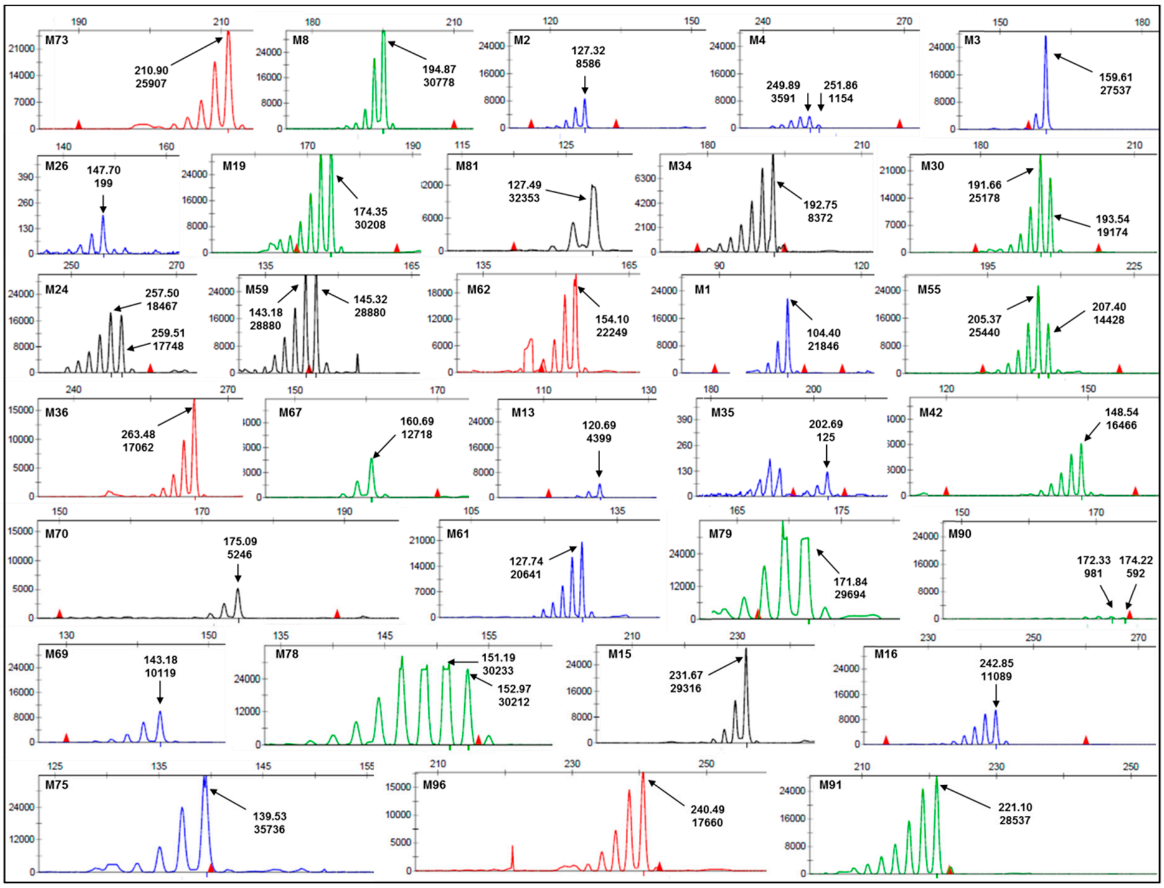

Figure S2. Chromatograms of the Short Tandem Repeat (STR) profile for the 31 variant markers in H9c2 cells. These chromatograms illustrate the amplification results of each dinucleotide repeat marker in the CellCheck™ Rat system. The peaks on the x-axis represent various allele sizes, with the peak height corresponding to the number of alleles. Each marker is assigned a number, and the allele sizes are measured in base pairs (bp).
