# Supplementary material for: Genetic and Molecular Characterization of H9c2 Rat Myoblast Cell Line
Source: Cells. 2025 Mar 28;14(7):502. doi: 10.3390/cells14070502 (PMC11988023; doi:10.3390/cells14070502)
Supplement: Supplementary file 1 [file cells-14-00502-s001.zip › Table S2.pdf]

Table S2. Molecular correlates of mRNA expression in H9c2 cells[1]

Transcript Id[1]	Gene Id	Gene	Gene description	TPM
ENSRNOT00000015179<br>ENSRNOT00000085126	ENSRNOG00000010765	*Vcl*	vinculin	212,536932<br>1,05275
ENSRNOT00000101345<br>ENSRNOT00000079085	ENSRNOG00000054172	*Ctnnb1*	catenin beta 1	148,736322<br>133,524514
ENSRNOT00000024430	ENSRNOG00000018087	*Vim*	vimentin	3019,935536
ENSRNOT00000105242<br>ENSRNOT00000042459<br>ENSRNOT00000080216<br>ENSRNOT00000116486	ENSRNOG00000034254	*Actb*	actin, beta	6847,442872<br>328,374415<br>0,831137<br>0,534563
ENSRNOT00000114387<br>ENSRNOT00000101412<br>ENSRNOT00000023014	*ENSRNOG00000016866*	*Fhl2*	four and a half LIM domains 2	77,911289<br>59,661397<br>45,106886
ENSRNOT00000110905<br>ENSRNOT00000057585<br>ENSRNOT00000019772<br>ENSRNOT00000102568	ENSRNOG00000014288	*Fn1*	fibronectin 1	1900,22356<br>1543,517182<br>238,42677<br>1,18139
ENSRNOT00000016423<br>ENSRNOT00000087619	ENSRNOG00000011292	*Col1a2*	collagen type I alpha 2 chain	11218,735077<br>1218,409973
ENSRNOT00000031164<br>ENSRNOT00000099722<br>ENSRNOT00000110155<br>ENSRNOT00000114705	ENSRNOG00000028629	*Akt1*	AKT serine/threonine kinase 1	213,839263<br>73,766958<br>59,571718<br>0,249067
ENSRNOT00000110990<br>ENSRNOT00000083156<br>ENSRNOT00000081355<br>ENSRNOT00000107560	ENSRNOG00000053468	*Tuba1a*	tubulin, alpha 1A	1549,529235<br>1425,203854<br>967,285871<br>267,308497
ENSRNOT00000083468	ENSRNOG00000058039	*Acta2*	actin alpha 2, smooth muscle	5216,58946
ENSRNOT00000028328	ENSRNOG00000020876	*Bax*	BCL2 associated X, apoptosis regulator	72,892241
ENSRNOT00000102631	ENSRNOG00000065095	*Cycs*	cytochrome c, somatic	35,86042
ENSRNOT00000004956	ENSRNOG00000003357	*Col3a1*	collagen type III alpha 1 chain	10639,937157
ENSRNOT00000086310	ENSRNOG00000059714	*Hsp90aa1*	heat shock protein 90 alpha family class A member 1	878,520549
ENSRNOT00000082010<br>ENSRNOT00000075886	ENSRNOG00000020513	*Becn1*	beclin 1	151,216261<br>1,303785
ENSRNOT00000001054<br>ENSRNOT00000100494	ENSRNOG00000000805	*Gja1*	gap junction protein, alpha 1	99,918567<br>17,905309
ENSRNOT00000050443<br>ENSRNOT00000110793<br>ENSRNOT00000041328<br>ENSRNOT00000114924	ENSRNOG00000018630	*LOC108351137*	glyceraldehyde-3-phosphate dehydrogenase	2955,510839<br>902,439425<br>147,63458<br>41,959217
ENSRNOT00000030919<br>ENSRNOT00000119519<br>ENSRNOT00000094152	ENSRNOG00000022619	*Fth1*	ferritin heavy chain 1	4501,99044<br>33,918056<br>8,764258

[1] The mRNA expression of selected genes that were also found at the protein level is depicted (cf. Figure 3).
